# Parallel/Opposed: Automated treatment planning systems should be designed to minimize human intervention

**DOI:** 10.1002/acm2.70618

**Published:** 2026-05-16

**Authors:** Carlos E. Cardenas, Joseph Harms, Dongxu Wang

**Affiliations:** ^1^ Department of Radiation Oncology The University of Alabama at Birmingham Birmingham Alabama USA; ^2^ Department of Radiation Oncology Washington University School of Medicine St. Louis Missouri USA; ^3^ Department of Medical Physics Memorial Sloan Kettering Cancer Center New York New York USA

**Keywords:** automation, treatment planning

## 1 INTRODUCTION

Dosimetric treatment planning in radiation therapy aims to maximize radiation dose to the lesion while minimizing radiation to the healthy tissues. Treatment planning is a multi‐variate process, involving the choice and combinations of beam energy, beam angle, field shape, spatial distribution of fluence, etc. Automation of this complex process has seen significant progress in the decades^1^ before the concept of artificial intelligence (AI) becomes popular. The fast adoption of AI in recent years has introduced new possibilities of treatment planning automation and raised a question: *should automated treatment planning systems be designed to minimize human intervention*, now that AI‐driven automation could potentially achieve that? Debating for this proposition is Dr. Carlos Cardenas from the University of Alabama at Birmingham, and debating against this proposition is Dr. Joseph Harms from Washington University in St. Louis.

**Carlos E. Cardenas, PhD**, is a board‐certified clinical medical physicist and associate professor at the University of Alabama at Birmingham. Dr. Cardenas serves as Director of Physics Research and Education and Director of Automated Treatment Planning within the Department of Radiation Oncology, as well as Director of AI Research and Development for the Marnix E. Heersink Institute for Biomedical Innovation. His areas of expertise include the development and clinical translation of artificial intelligence systems to automate radiotherapy treatment planning workflows, as well as the implementation of CBCT‐based adaptive radiotherapy. He also leads a research lab focused on artificial intelligence and adaptive radiotherapy, with an emphasis on developing and translating innovative technologies into clinical practice. Dr. Cardenas has co‐authored more than 100 peer‐reviewed manuscripts in these areas. He currently serves on several American Association of Physicists in Medicine (AAPM) Task Groups (TG263U1, TG384, TG395, TG424, and TG455) and co‐chairs the American Society for Radiation Oncology (ASTRO)’s Artificial Intelligence Task Force.

**Joseph Harms, PhD**, is a board‐certified clinical medical physicist and assistant professor at Washington University School of Medicine. Dr. Harms completed an MS in Medical Physics and PhD in Nuclear Engineering, both from the Georgia Institute of Technology, prior to completing a Therapeutic Medical Physics Residency at Emory University. His areas of expertise within radiation therapy include proton therapy, online adaptive radiation therapy, and automated treatment planning. Dr. Harms’ research focuses on incorporation of novel technology into the clinic, from concept development to clinical deployment, to improve the safety and efficacy of radiation therapy. He has overseen clinical implementation of various novel treatment technologies, including advanced 6‐s CBCT imaging and optical surface monitoring for adaptive and proton radiation therapies. Recently, he has led a Radiation Oncology Institute‐funded project focusing on radioluminescent imaging for real‐time quality assurance of proton FLASH beams. Dr. Harms has been recognized on Georgia Tech's 40 Under 40 for his contributions as an innovator in the field of Radiation Oncology. He serves as an associate section editor for the International Journal of Radiation Oncology, Biology, Physics, and serves as a regular reviewer for several journals, including Medical Physics, Physics in Medicine and Biology, and Practical Radiation Oncology.

## 2 OPENING STATEMENTS

### 2.1 Opening statement from Dr. Carlos Cardenas, for the proposition

The proposition that automated treatment planning systems should be designed to minimize human intervention can be defended on principle, but it is strongest when grounded in real‐world data. The evidence shows two consistent patterns: (1) manual planning produces substantial and clinically meaningful variability, even under controlled conditions; and (2) well‐designed automation can improve consistency and often reduce planning time without degrading plan acceptability.^2^, ^3^, ^4^

“Minimize” should be read as a system design requirement, not as an argument for unsupervised autonomy. In mature implementations, humans still define the prescription intent and constraints, and clinical teams still approve the plan. What is minimized is repeated trial‑and‑error (tuning objectives, weights, and planning structures) for each individual case. Instead, clinical intent is encoded up front into templates and scorecards so that most cases run end‑to‑end with only exception handling.^5^

The case for this design begins with a hard truth that human planning output varies widely. Nelms *et al* ^6^ previously showed in an inter‐institutional study that even with ambiguity removed, there was wide variation in plan quality amongst different planners; the variation was not explained by the treatment planning system, delivery modality, or planner demographics. This is the technical and ethical foundation for the “should.” Variation in plan quality means variation in organ sparing and trade‑offs; it is difficult to justify a workflow that expects variation when standardization is achievable.

Multiple clinical studies show that automation can do exactly that. In prostate VMAT, Scaggion *et al* ^2^ trained a dose‐histogram estimation model on 70 clinical plans and asked seven planners of varying experience to plan 15 patients with and without knowledge‐based planning (KBP) assistance. KBP plans improved rectum/femoral head sparing, increased overall plan quality for many patients, and, critically, reduced inter‑planner variability: the interquartile range of overall plan quality metric was approximately halved! This is the operational definition of “minimize intervention” resulting in fewer human‐dependent degrees of freedom that drive quality spread.

Head and neck data reinforce the point. Hansen *et al* ^7^ prospectively generated both manual and auto‑plans for 30 consecutive patients and performed a blinded clinical selection. Auto‑plans were selected for treatment for 29/30 patients; organ‑at‑risk doses were significantly reduced by 0.5–6.5 Gy while maintaining target coverage, and average active operator time dropped from 64 min to roughly half that with automated planning. In a separate multi‑institution blinded physician review, Olanrewaju *et al* ^8^ compared automated plans with delivered clinical plans in 50 head and neck patients; 88% of auto‐plans and 78% of clinical plans were judged usable “as is”, and reviewers more often preferred the auto‐plan.

It is important to note that automation is also an economic and capacity strategy. Vanderstraeten *et al* ^4^ compared manual and automated lung SBRT planning in 56 patients and found a 77.3% reduction in average optimization time while preserving target coverage; >75% of automated plans were clinically acceptable without further fine‑tuning. This matters because staffing constraints are real. A national staffing survey reported vacancy rates of 10.7% in radiation therapy and 11.4% in medical dosimetry (unfilled FTE positions).^9^ Given current staffing shortages (including those in medical physics), reclaiming skilled planning time is not a marginal benefit as it directly improves access and throughput. More importantly, speed matters clinically because delays matter in cancer care. In a systematic review/meta‑analysis covering 34 studies and 1,272,681 patients, Hanna *et al* ^10^ found that each four‑week delay in radical radiotherapy for head and neck cancer was associated with increased mortality risk (hazard ratio 1.09 per four weeks). Automated planning will not fix every delay driver, but it is one of the few system levers that can reduce turnaround time without depending on heroics or overtime.

Finally, “minimize intervention” is compatible with safety only if automated systems are designed for safety explicitly. Risk‑informed process mapping and FMEA approaches, as laid out in Task Group 100,^11^ emphasize that many radiotherapy errors arise from workflow and process failures rather than device failures. A joint guideline from ESTRO and AAPM on AI in radiotherapy likewise emphasizes development, clinical validation, reporting, and practical commissioning considerations as part of governance and lifecycle QA.^12^ Within that framing, automation can add safety barriers. For example, Gronberg *et al* ^13^ used deep learning dose prediction for automated, individualized plan QA and reported that physicians’ improvement requests can be highly variable (83% of physician‐flagged organs were flagged by only one of three physicians), motivating standardized automated QA to normalize review.

For a technology that is already producing clinically acceptable plans without edits in large fractions of cases, while improving consistency and reducing planning time, the correct design target is clear: automated treatment planning systems should be built to minimize routine human intervention in plan creation, with human effort focused on clinical judgment, quality assurance, and final plan approval.

### 2.2 Opening statement from Dr. Joseph Harms, against the proposition

There has been no shortage of interest within the medical physics community in auto‐planning. A query for “automated radiation treatment planning” in Google Scholar with a filter for papers since 2018 generated over 18,000 results. The advance of artificial intelligence (AI) has brought the promise of auto‐planning to a new height; as this debate proposition states, perhaps auto‐planning methods, especially those driven by AI, would no longer need human intervention. While the topic has been of great interest to our field, prospective data on the integration of AI‐driven auto‐planning solutions remain relatively sparse,^12^, ^14^, ^15^ though more data is becoming regularly available. As stated by Nguyen *et al*,^1^ many research studies fail to yield clinical results simply because practical challenges of clinical integration are often overlooked by researchers. Of the limited prospective studies on clinical implementation of automated treatment planning, multiple have found a several percentages points drop in physician preference for AI‐generated plans between pre‐clinical testing and clinical adoption.^15^, ^16^, ^17^ *If we want AI to change our field, we need to design automated planning systems to augment the skills of the treatment planning team, not to replace them*.

*Designing automated treatment planning systems with humans‐in‐the‐loop incentivizes planners to learn about the system*. Too often, we as physicists aim to design these systems without input from members of the team. Then, as models are released into the clinic, planners find them difficult to work with, and because of this, they may go neglected.^18^ Without regular interaction with the automated planning algorithm, there is limited opportunity for treatment planners to build familiarity with the nuances of the algorithm. In a survey of medical dosimetrists in 2020–2021, 62% of respondents found that difficulty in modifying automated treatment plans limited their usefulness.^18^ One possible contributor to this implementation barrier is education, and for many people, the best way to learn is through practice. When systems are designed to circumvent the planning team, there is no opportunity to learn through interaction. The autoplanner produces a result, and if there is no straightforward way to modify the plan, it is scrapped by the planner.

Integral to building interactive systems is designing them so that plans can be adjusted if needed, and those adjustments are best tuned by experienced planners. In a prospective implementation study at my prior institution,^19^ we found that automatically generated plans, using RapidPlan, alone showed similar quality to manual plans. However, when manual intervention was incorporated with the autoplanner during optimization, plan quality overall improved as compared to manual planning alone. In a prospective study using an autoplanner for breast radiation therapy, Bakx *et al* ^17^ found that while 74% of AI‐generated RT plans met dosimetric goals, small manual adjustments brought the passing rate to 86%. Notably, in 35% of AI‐generated plans that met all goals initially, dosimetrists still adjusted the autoplans. While evaluating dose metrics is inherently quantitative, there is still a qualitative portion to plan review that is critical, and taking planners out of the loop removes that qualitative element.

A commonly identified barrier to adoption of automated solutions in healthcare is a lack of interpretability and explainability.^20^ Systems need to be interpretable so we can understand when they fail and explainable so we can set reasonable expectations.^12^ *Augmentation‐designed systems promote accountability*. As many of us have learned from incorporating AI‐based contouring in the clinic, it is easy for planners, physicists, or physicians to say, “the algorithm made the mistake, not me.” In the end, it does not matter who makes mistakes if they reach the patient. When humans are forced to interact with the planning system, they inherently take a more detailed look at the plans it produces. If dosimetrists or physicists are reviewing the plan with the intent to look for areas of improvement, the scope of their review will be more detailed than just reviewing. Additionally, if the system is built to work with the team, there is higher potential for continuous model improvement. Planners will more readily notice systematic differences between the plans produced by the model and their a priori knowledge.

While I believe that artificial intelligence will change the landscape of radiation oncology, it will not happen overnight. Rather, the incorporation of AI into our clinical lives will continue as a slow creep to allow all members of the treatment team time to familiarize themselves with how AI systems may differ from historical practices. This is paramount to safe clinical implementation of autoplanning. Conroy *et al* ^15^ summarized a key component to my argument best: “For healthcare applications poor explainability can undermine the trust in the output of AI and act as a barrier to widespread clinical adoption. When the end user cannot easily understand the ‘reasoning’ behind an output trust in the AI algorithm decreases.” For autoplanning systems to be explainable, they need to be interacted with on a regular basis, and humans need to be in the loop.

## 3 REBUTTALS

### 3.1 Rebuttal from Dr. Carlos Cardenas, for the proposition

Dr. Harms is right to emphasize integration realities.^14^ Workflows fail clinically not because an algorithm cannot generate a plan, but because the system is hard to deploy, hard to trust, and hard to use. Yet those points argue for the proposition, not against it. If practical integration is commonly overlooked, then planning systems should be engineered so routine success does not depend on repeated manual tuning of objectives and weights.^1^

His own adoption argument makes this clearer. In the JACMP dosimetrist survey^18^ he cites, 62% reported that difficulty modifying an automated plan limited usefulness. That finding is not an argument to require frequent human intervention, it is evidence that needing to intervene often is a design failure mode. The correct solution is to improve first‐pass plan quality and make the intervention pathway deliberate, guided, and efficient (targeted edits only when an automated scorecard/QA flags an outlier). Likewise, the breast example (Bakx *et al*)^17^ he cites can be read as support for minimization: 37/50 plans (74%) met all clinical goals without adjustments, and with small manual adjustments this increased to 86%. That is exactly the design objective we are advocating: make the default pathway require no edits for most patients, and reserve human intervention for the minority that truly need it. Moreover, the observation that dosimetrists still adjust some plans that already meet goals highlights why routine “tweaking” is a pathway to variability and rework and not necessarily leading to better care.

Finally, the claim that prospective/real‐world evidence is sparse is increasingly dated. Blinded prospective and multi‐institution studies show that automated plans can be selected for treatment at high rates (e.g., 29/30 in Hansen *et al* ^7^) and judged usable “as is” at high rates (e.g., 88% in Olanrewaju *et al* ^8^). These are not “replace the team” studies, they are “first‐pass success + clinician review” studies, consistent with minimizing routine intervention while preserving accountability. Importantly, much of this evidence was generated using earlier‐generation, largely template‐ and rule‐driven automation and optimization engines. It is reasonable to view these results as a conservative baseline. Newer optimization algorithms and emerging agentic AI approaches are actively being explored and could further increase first‐pass plan quality and robustness while keeping clinicians firmly in the approval loop.^21^

Taken together, the evidence supports the clear direction that automated treatment planning systems should be designed for first‐pass success, so routine cases require minimal manual intervention, with human expertise reserved for clinical judgment, exceptions, and final approval. That design choice raises the floor on quality and consistency, and it protects capacity in a workforce environment where time is the limiting resource. It is important to emphasize that the take home message is not ‘all hands‐off’; the take home message needs to be properly framed as ‘automation with guardrails.’ Minimization is only safe when it is paired with disciplined implementation: risk‐based workflow analysis (e.g., TG‐100‐style process mapping and FMEA),^11^, ^22^ explicit acceptance criteria and automated plan QA/outlier detection, team training on known failure modes, and ongoing validation and monitoring as systems and models evolve.^23^ Done rigorously, automation reduces variability and delays without sacrificing accountability, but when deployed without adequate commissioning, monitoring, and governance, it simply allows mistakes to propagate faster.

### 3.2 Rebuttal from Dr. Joseph Harms, against the proposition

Dr. Cardenas’ opening argument is centered around two main theses:

(1) Manual planning produces substantial and clinically meaningful variability
(2) Well‐designed automation can improve consistency and often reduce planning time without degrading plan acceptability

Both statements align well with my personal experience in the clinic and on my thoughts as a clinical scientist. However, designing systems to minimize human intervention is not a necessary condition for these statements to be true.

One other sentence from Dr. Cardenas’ opening statement stands out: “Minimize should be read as a system design requirement, not as an argument for unsupervised autonomy.” I think anyone reading this article will agree that unsupervised autonomy for autoplanning systems is not a desirable outcome. However, my argument against the proposition is focused on the concept that supervision of these automated treatment planning systems needs to be performed by experts with clinical experience. As noted by Callens *et al* ^23^ in a recent review paper on automated treatment planning, the clinical integration of automation should be accompanied by transparency regarding the applications and limitations of the process. Autoplanning systems have become an integral part of treatment planning workflows for online adaptive radiation therapy, which has grown in utilization over recent years. Safety analyses in this space have deemed oversight from individuals with the necessary expertise critical for safe clinical implementation.^24^, ^25^ Building that expertise requires understanding failure modes and how to troubleshoot, and that experience is best built through human‐centric design. Designing automated treatment planning systems that augment current clinical experience democratizes the clinical implementation of these systems and opens the evaluation up to end users. This approach has the added benefit of offloading continuous maintenance and quality assurance to member of the treatment team rather than relying solely on system designers.

Human planning output varies widely. Several studies have shown that autoplanning solutions such as knowledge‐based planning can reduce this variation, flag outliers, and improve overall plan quality.^26^ In a human‐centric system design, the goal is for utilization of autoplanning to raise the minimum threshold for plan quality, but the system should be designed such that plans can either be improved in terms of overall plan quality. In other words, *autoplanning models should raise the floor for plan quality, not set the ceiling*. An added benefit of designing models to augment human planners, rather than replace them, is to allow for slight model modifications to tailor to patient‐specific criteria. For example, in the case of lung irradiation for patients with interstitial lung disease, it is reasonable to expect that reducing lung dose may be more important than other typical organs‐at‐risk. In autoplanning systems designed for minimal human intervention, it may be difficult or impractical to include this information in the treatment plan. However, if autoplanning relies on some level of human intervention, the plan optimization goals can be modified slightly to shift overall importance to the healthy lung tissue.

In the era of patient‐centered care, automated systems need to be dynamic. Centering these systems around humans‐in‐the‐loop is the safest way to bring automation into daily clinical practice.

## AUTHOR CONTRIBUTIONS

Carlos E. Cardenas, Joseph Harms initiated the discussion and drafted the main text. Dongxu Wang provided critical edits and comments.

## CONFLICT OF INTEREST STATEMENT

Carlos E. Cardenas and Joseph Harms declare no conflicts of interest. Dongxu Wang reports receiving a licensing fee payment from Ion Beam Applications, S.A., outside of this work.
